# Assessment of Plaque Characteristics by Contrast-Enhanced Ultrasound and Stent Restenosis following Carotid Artery Stenting: A Retrospective Study

**DOI:** 10.3390/medicina60050836

**Published:** 2024-05-20

**Authors:** Agnė Gimžauskaitė, Donatas Inčiūra, Gintautė Diringytė, Saulius Lukoševičius, Rytis Kaupas, Andrius Pranculis, Aistė Mačiulaitytė, Algidas Basevičius, Milda Kuprytė, Edgaras Stankevičius, Jurgita Plisienė

**Affiliations:** 1 Department of Cardiac, Thoracic and Vascular Surgery, Medical Academy, Lithuanian University of Health Sciences, LT-50161 Kaunas, Lithuania; donatas.inciura@lsmu.lt (D.I.); aiste.maciulyte@stud.lsmu.lt (A.M.); 2 Faculty of Medicine, Medical Academy, Lithuanian University of Health Sciences, LT-44307 Kaunas, Lithuania; gintaute.diringyte@stud.lsmu.lt; 3 Department of Radiology, Medical Academy, Lithuanian University of Health Sciences, LT-50161 Kaunas, Lithuania; saulius.lukosevicius@lsmu.lt (S.L.); rytis.kaupas@lsmu.lt (R.K.); andrius.pranculis@kaunoklinikos.lt (A.P.); algidas.basevicius@lsmu.lt (A.B.); 4 Department of Pathological Anatomy, Lithuanian University of Health Sciences, LT-50161 Kaunas, Lithuania; milda.kupryte@lsmu.lt; 5 Institute of Physiology and Pharmacology, Lithuanian University of Health Sciences, LT-50161 Kaunas, Lithuania; edgaras.stankevicius@lsmu.lt; 6 Department of Cardiology Medical Academy, Lithuanian University of Health Sciences, LT-50161 Kaunas, Lithuania; jurgita.plisiene@lsmu.lt

**Keywords:** contrast-enhanced ultrasound, carotid stenting, carotid stent restenosis, carotid artery disease

## Abstract

*Background and objective:* carotid artery stenosis contributes significantly to ischemic strokes, with management options including carotid endarterectomy (CEA) and carotid artery stenting (CAS) ischemic stroke risk can be reduced. Controversies persist regarding their efficacy and factors influencing complications, and understanding the relationship between atherosclerotic plaque characteristics and stent restenosis after CAS is crucial. *Methods:* we conducted a retrospective study involving 221 patients who underwent CAS for symptomatic or asymptomatic carotid artery stenosis. Comprehensive assessments of plaque morphology were performed using contrast-enhanced ultrasound (CEUS) before CAS. Patient demographics, including smoking status and diabetes, were also recorded. Stent restenosis was diagnosed using various imaging modalities, including ultrasound, angiography, and digital subtraction angiography (DSA). *Results:* plaque analysis using CEUS revealed a significant association between plaque grade and restenosis incidence (*p* < 0.001), particularly with grade 0 (11.1%) and grade 2 plaques (66.7%). Smoking was notably associated with plaque vascularization and restenosis (*p* < 0.001), while diabetes did not significantly impact plaque characteristics or restenosis risk (*p* > 0.05). The mean duration of restenosis was 17.67 months. Stenting was the most frequent treatment modality for restenosis (70.6%). However, no significant relationship was found between restenosis type and plaque morphology (*p* = 0.268). Furthermore, while no clear relationship was observed between plaque morphology and the type of restenosis, our findings underscored the importance of plaque characterization in predicting post-CAS outcomes. *Conclusions:* this study highlights the utility of CEUS in predicting stent restenosis following CAS. There was a significant association between stent restenosis within 12–24 months after the carotid stenting procedure and an elevated grade of plaque vascularization. Moreover, one of the main factors possibly determining the grade of plaque vascularization was smoking. Further research is warranted to elucidate the underlying mechanisms and refine risk stratification in this patient population.

## 1. Introduction

Stroke is the second most common cause of death and a leading cause of adult disability in the European Union [1]. As life expectancy increases, stroke events and their long-term consequences, as well as the corresponding costs, are expected to increase dramatically [2]. Carotid atherosclerotic disease is a significant health concern that contributes to about 20% of cases of cerebral ischemia [3], caused by either reduced blood flow to the central nervous system due to carotid stenosis or by the formation of clots on the surface of the plaque and subsequent arterio-arterial embolization [4]. Traditionally, patient management and risk stratification have relied on the presence of symptoms and the degree of stenosis—both are linked to the occurrence of stroke events [5]. After evaluating the factors, physicians choose an interventional treatment method for a patient: endarterectomy or stenting.

In contemporary times, with the advancement of scientific understanding enabling deeper insights into various processes, it becomes imperative to acknowledge the dynamic nature of atherosclerotic plaque as a biologically active entity. The traditional view of atherosclerosis as a pathological lipid deposition within the artery wall has been redefined by a more complex concept of an ongoing inflammatory process [6]. Many molecular players have a role in the atherosclerotic process: inflammatory cytokines, markers of oxidative stress, growth factors, apoptosis mediators, and mediators of vascular tone. The relationship between the stent and the biologically active structure (atherosclerotic plaque) can lead to stent restenosis. Randomized controlled trials have reported that carotid endarterectomy (CEA) and carotid artery stenting (CAS) yield comparable long-term results in reducing ischemic stroke risk over 10 years [7]. However, there is a long-standing debate about which treatment is more effective and which factors must be considered to predict potential complications. The 10-year data of the CREST trial have revealed no discernible difference in restenosis or the requirement for revascularization between CEA and CAS [8]. CAS is linked to a higher 30-day minor stroke risk compared to CEA. Restenosis and occlusion after CEA and CAS have been reported to have a low incidence and no difference in two years. A restenosis rate of >70% by duplex criteria at two years was 6% in CAS and 6.3% in CEA [9]. A recent systematic review and meta-analysis has indicated that the incidence of restenosis exceeding 70% was 5.8% following CEA (median follow-up of 47 months) and 10% following CAS (median follow-up of 62 months). Among CAS patients with untreated asymptomatic restenosis exceeding 70%, the rate of ipsilateral stroke remained extremely low, at 0.8% over 50 months. In contrast, CEA patients with untreated asymptomatic restenosis exceeding 70% had a higher rate of ipsilateral stroke, albeit still relatively modest at 5% within 37 months [10].

After follow-ups for six months to 2 years, the incidence of in-stent restenosis (ISR) has been found to range from 3.3% to 21% [11,12,13].

Risk factors for CAS restenosis include female sex, dyslipidemia, and diabetes [14]. However, a gap exists where patients without these risk factors still experience restenosis, suggesting a potential link to atherosclerotic plaque morphology. The atherosclerotic plaque should be considered as a constantly changing structure [15]. Multiple studies have shown a link between intraplaque hemorrhage and brain ischemic events [16,17,18,19]. Intraplaque hemorrhage can result from the rupture of fragile microvessels within the atherosclerotic plaque, which are formed due to local inflammation-induced neoangiogenesis. These vessels are characterized by being immature and inadequately structured, with leaky endothelial junctions that increase their susceptibility to rupture. Presence of immature vessels—neoangiogenesis—and active inflammatory processes within the plaque might be an important factor when considering the stent restenosis—there is an active interaction between the plaque and the stent.

Contrast-enhanced ultrasound (CEUS) has emerged as a valuable tool in characterizing plaques, aiding the understanding of their role in stent restenosis mechanisms. Among the array of available imaging modalities, CEUS is emerging as a readily accessible option, providing valuable insights into plaque morphology and neovascularization patterns. Previously, this technique was used to assess plaque vulnerability and predict recurrent strokes. Therefore, the idea that atherosclerotic lesion neovascularization is a risk factor for restenosis after CAS could lead to acquiring new scientific facts. In our study, contrast-enhanced ultrasound scans were conducted on patients before CAS. Carotid atherosclerotic lesions were characterized using the classification by Nakamura et al. which includes categories G0, G1, and G2 [20]. The primary objective was to ascertain whether the nature of the atherosclerotic lesion and the degree of its neovascularization, as detected on CEUS, were associated with stent restenosis.

## 2. Materials and Methods

### 2.1. Patients

A total of 221 patients were selected and analyzed in this study. A total of 68.8% (*n* = 152) were men and 31.2% (*n* = 69) were women.

Inclusion criteria:

- Transient ischemic attacks and ACI stenosis of ≥50%
- Stroke and carotid stenosis ≥ 50%
- Signed informed consent

Exclusion criteria:

- Any known allergy to ultrasound contrast agent (SonoVue)
- Right-to-left shunts
- Severe pulmonary hypertension (pulmonary artery pressure > 90 mmHg)
- Uncontrolled systemic hypertension
- Adult respiratory distress syndrome
- Severe calcification of atherosclerotic lesion (based on CT)
- Tortuosity of carotid vessel
- Near occlusion cases (with 99% stenosis)
- Very calcified carotid lesions

Dyslipidemia was defined as an increase in laboratory markers reaching total cholesterol > 5.2 mmol/L (200 mg/dL), LDL > 3.4 mmol/L (130 mg/dL), HDL < 0.9 mmol/L (35 mg/dL), or triglycerides > 1.7 mmol/L (150 mg/dL). All patients who had a diabetes diagnosis had already been prescribed sugar-lowering drugs and that was considered (based on medical history) as diabetes, additional tests during the study were not performed. Patients who were diagnosed with diabetes during the follow-up period were also considered to be diabetic. Smoking status was defined as being a current or former smoker.

### 2.2. Ultrasound Characteristics

For all patients, CEUS was performed using a Philips Epiq 7 G ultrasound machine with a pulse inversion mode, software version 6.0.0., and a linear transducer with a frequency range of 3–11 MHz, MI < 0.2.

### 2.3. Ultrasound Contrast Agent

The contrast agent comprises gas bubbles encased within a lipid or protein shell. These microspheres are exceptionally diminutive and can traverse the entirety of the circulatory system, mirroring the blood’s path. The dimensions of SonoVue bubbles measure 6 μm. In Lithuania, SonoVue, a product by Bracco, has received approval. It encompasses sulfur hexafluoride (elegas, SF6-enflurane) in conjunction with phospholipid stabilizers. SF6-enflurane boasts superior dielectric properties, lacks color, taste, and odor, and is approximately six times denser than air. Under normal conditions, it poses no toxicity risk. The ultrasound ruptures the microspheres within the body, allowing other gas bubbles to course through the bloodstream, with larger quantities being exhaled. Metabolism of the lipid shells occurs in the liver, ultimately being expelled through the bile.

The contrast agent was administered via an intravenous catheter inserted into the cubital vein. We followed the conventional method, administering a 2.5 mL bolus of the contrast agent and 5 mL of saline. Notably, no patients exhibited contraindications for the use of SonoVue.

### 2.4. Principles of Contrast Ultrasound Based on Contrast Agent Pharmacokinetics

**Figure 1:**
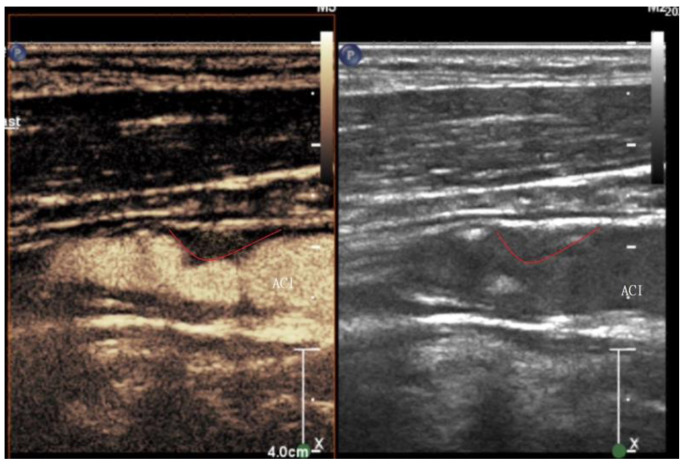
Representative CEUS (**left**) and grayscale ultrasound (**right**) images of a grade 2 plaque. The red curved line shows the plaque margins.

**Figure 2:**
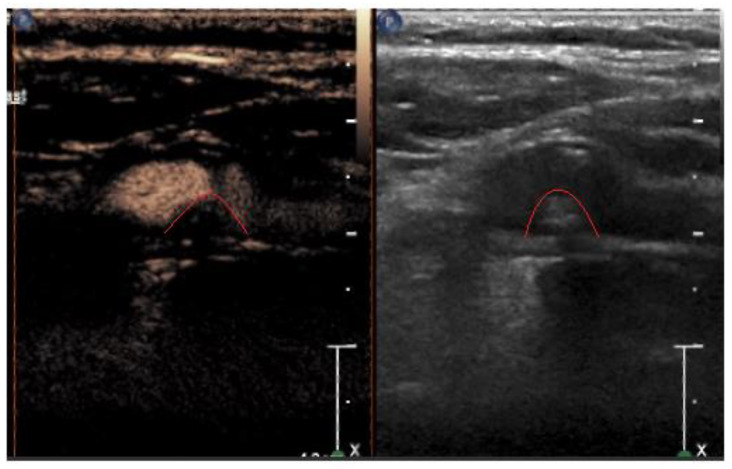
Representative CEUS (**left**) and grayscale ultrasound (**right**) images of a grade 1 plaque. The red curved line shows the plaque margins.

**Figure 3:**
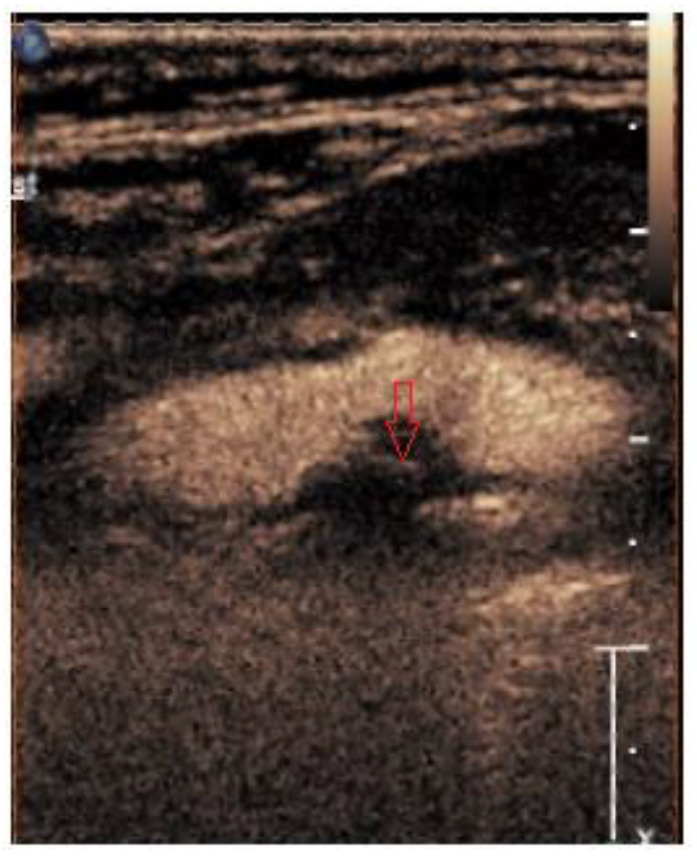
Representative CEUS image showing neovascularization with a red arrow.

Microspheres exhibit a varying behavior in response to the mechanical index of the ultrasound beam, reflecting the cavitation effect. The mechanical index also signifies the degree of negative acoustic pressure within the ultrasound field, denoting the highest-pressure pulse amplitude within the tissue during ultrasound procedures. Our examination used a linear imaging probe featuring a low mechanical index to prevent microsphere rupture. Our focus was primarily on the arterial and venous phases when categorizing plaques as grades G0–2 (according to the classification by Nakamura et al.) [20]. The visual score method was employed. Intraplaque neovascularization was identified based on the presence of microbubbles within the plaque, with grading encompassing G0 (not visible), G1 (moderate), and G2 (intensive microbubbles). Representative images of intraplaque neovascularization of different grades are shown in Figure 1, Figure 2 and Figure 3.

### 2.5. Criteria for Carotid Stenting

Before the CAS procedure, assessment of carotid atherosclerotic lesion morphology with CEUS was conducted for all patients. CAS was performed for the patients with transient ischemic attacks (TIAs) or for patients who had suffered a stroke within the past six months and had a carotid stenosis ≥ 50% [21]. For TIAs, we used a time-based definition. All the patients from the neurological department with the indications for CAS were transferred to interventional radiologists to perform CAS.

### 2.6. Carotid Artery Stenting (CAS)

Before the planned interventions, dual oral antiplatelet therapy was prescribed: clopidogrel 75 mg and aspirin 75–100 mg per day. We started treatment at least 3 days before the stenting procedure. In urgent or emergent interventions, a single oral loading dose of 300 mg clopidogrel and 300 mg aspirin was given, followed by dual antiplatelet therapy of 75 mg clopidogrel plus 75–100 mg aspirin daily. We recommended a regimen including dual antiplatelet therapy (aspirin + clopidogrel) during the three months period after CAS and then reverted to monotherapy (aspirin 75 mg daily). After the procedure, we continued the previous treatment of comorbidities. Additionally, we added recommendations to correct any abnormalities in the lipid profile and encouraged smoking cessation for better health outcomes. Usually, patients had already been prescribed statins. If not, we prescribed statins right away.

The procedure was performed with local anesthesia.

Stenting of the carotid artery was started by injecting Heparin 80 IU/kg i/v and 0.6–1.2 mL atropine (glycopyrrolate) to prevent hypotension, bradycardia, or asystole. Routinely, we punctured the femoral or brachial artery using Seldinger’s technique and inserted the introducer of 6 F. The lesion was crossed with 0.014 in. wire. Predilatation was performed with a 2-to-3 mm balloon. The stent was placed across the lesion and deployed. A repeat arteriogram was performed. Any residual stenosis exceeding 30% was treated with balloon angioplasty. Sheaths and wires were removed, and an access site closure device was deployed. We did not use any distal embolization protective devices. After stenting, active patient monitoring for at least 6 h and monitoring of BP and HR as needed should be ensured in order to avoid hemodynamic instability. In our center, we use a few types of stents.

This study analyzed cases where the same stents were used for primary stenting. The patients who had very calcified carotid lesions were excluded from this sample because of the restenosis risk. There were seven patients who were excluded: two patients had tortuosity of the artery, three had near occlusion (stenosis of 99%), and two patients had very calcified lesions.

### 2.7. Stent Restenosis

**Figure 4:**
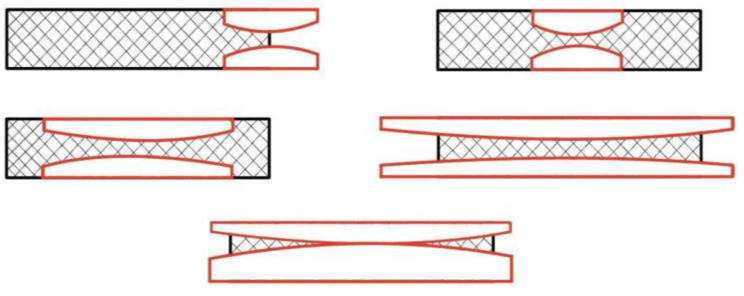
Types of restenosis after the stenting procedure [22].

There are several types of restenosis classified (Figure 4):

- Type I (focal end-stent group): lesions are 10 mm long and are positioned at the proximal or distal margin (but not both) of the stent. Lesions 10 mm long at both ends of the stent are defined as type I—multifocal end-stents.
- Type II (focal intra-stent group): lesions are 10 mm long and are confined within the stent(s) without extending outside the margins. Two or more discrete lesions 10 mm in length located within the stent are defined as type II—multifocal intra-stent.
- Type III (diffuse intra-stent group): lesions are 10 mm long and are confined within the stent(s) without extending outside the margins.
- Type IV (diffuse proliferative group): lesions are 10 mm long and extend beyond the margin(s) of the stent(s).
- Type V (occlusion group): lesions have no prograde flow and no lumen has been identified [22].

Another potential contributor to carotid in-stent restenosis (Figure 4) is the insufficient expansion of the initial stent, possibly due to external compression arising from significant calcification. The cases where insufficient expansion was seen were excluded from the study. It is worth mentioning that all those highly calcified lesions had no signs of neovascularization (detected with CEUS).

### 2.8. Diagnosis of Stent Restenosis

In our center, we perform ultrasound follow-ups every 2 months in the first year and then only every 6 months after stent placement. If there is any suspicion of stent restenosis, the patient is subjected to DSA. If a patient comes back with any neurological symptoms (suspicious of cerebral ischemia), we perform DSA as the primary diagnostic tool.

### 2.9. Treatment Strategy

Balloons, stents, and drugs were used to treat restenosis. If stenosis was less than 50%, and the patient was ipsilaterally asymptomatic, those cases were left for the best medical treatment strategy. If stenosis was more than 50%, then it was treated with repeat CAS (rCAS), followed by percutaneous transluminal angioplasty (PTA).

### 2.10. Ethical Approval

This study was conducted with an ethical approval from Kaunas Regional Biomedical Research Ethics Committee (Approval No. BE-2-102, dated 13 November 2020). After receiving comprehensive information about the study, all the participating patients provided the informed consent.

### 2.11. Statistical Analysis

Statistical analysis was performed using IBM SPSS 23.0 (Statistical Package for the Social Sciences). Categorical data were expressed as numbers with percentages, while continuous data as means or medians. The chi-square criterion was used to test the hypothesis about the association between two characteristics. The non-parametric Mann–Whitney U test to compare two independent samples was used. Kendall correlation analysis was performed. A correlation was considered to be weak if τ was <0.3, moderate if 0.3 < τ < 0.7, and strong if τ > 0.7. Post hoc power analysis showed a statistical power of >0.8. The significance level α = 0.05 was chosen for statistical hypothesis testing. Differences were considered statistically significant if *p* < 0.05.

## 3. Results

### 3.1. Characteristics of the Study Population

The mean age of all patients was 71.7 years. Table 1 presents the characteristics of the overall study population. In our study, the patients were divided into two groups: stented patients without restenosis and stented patients with restenosis. Statistical analysis using the chi-square test revealed no significant sex-specific distribution for the two patient groups (*p* = 0.464). The Mann–Whitney U test showed no significant difference in the median age of patients with and without restenosis (70.50 and 72.0 years, respectively, *p* = 0.529).

Stented patients without restenosis accounted for 91.9% (*n* = 203) and stented patients with restenosis accounted for 8.1% (*n* = 18) (Table 2).

### 3.2. Patients’ Distribution by Plaque Grade

Grade 0 intraplaque neovascularization detected by CEUS was identified in 51.6% (*n* = 114) of patients, grade 1 in 30.8% (*n* = 68), and grade 2 in 17.6% (*n* = 39). Notably, a statistically significant association was established between plaque grade and both stented patient groups: with restenosis and without restenosis (*p* < 0.001).

The distribution of patients without and with restenosis by plaque grade is shown in Table 3. A statistically significant difference between both patient groups and the grade of plaques was found only in grade 0 (*p* < 0.001) and grade 2 (*p* < 0.001), while there was no statistically significant difference in grade 1 (*p* > 0.05). We randomly selected 69 patients out of 203 to test for reliability. In a random sample, the percentage of stented patients without and with restenosis was 31.2% (*n* = 69) and 8.1% (*n* = 18), respectively. The random sample statistical analysis revealed significant differences in plaque grade between the two patient groups (*p* < 0.001). The distribution of grade 0 plaque in the stented group without restenosis reached 62.3% and stented with restenosis reached 11.1%. Distribution of grade 1 plaques: without restenosis—29.0%, with restenosis—22.2%. Grade 2 plaque distribution: without restenosis—8.7%, with restenosis—66.7%.

### 3.3. Distribution of Smoking and Diabetes in the Patient Groups

Upon data analysis, it was determined that 89.6% (*n* = 198) of patients were non-smokers and the percentage of smokers was 8.1% (*n* = 18). The chi-square test indicated a notable association between smoking and both patient groups: stented patients without restenosis (5.5%) and stented patients with restenosis (43.8%) (χ^2^ = 28.375, df = 1, *p* < 0.001). The risk estimate test showed a 13.36% chance of developing restenosis due to smoking. A random sample confirmed that the prevalence of smoking exhibited a statistically significant difference between patients without and with restenosis (4.3% vs. 43.8%). In addition, 76.9% (*n* = 170) of stented patients without restenosis and 20.8% (*n* = 46) with restenosis had diabetes (*p* > 0.05).

### 3.4. Comparison of Plaque Grades and Smoking/Diabetes

Statistical analysis revealed a significant difference between the two patient groups with respect to the grades of plaques and smoking (*p* = 0.007) (Table 4). However, by dividing all smoking patients into two separate groups of smokers, stented with restenosis and stented without restenosis, we got statistically insignificant differences (*p* > 0.05). The risk estimate test showed a 1.58% chance of developing grade 0 and 1 plaques due to smoking. The results showed no statistically significant difference between the two patient groups in terms of plaque grade and diabetes, stented with restenosis and stented without restenosis (*p* = 0.077).

### 3.5. Diagnosis of Restenosis: US, Angiography

The mean duration of restenosis was 17.67 months. The frequency of angiography was 61.1% (*n* = 11), and the frequency of US was 33.3% (*n* = 6).

### 3.6. Treatment of Restenosis

Balloons, stents, and drugs were used to treat restenosis. The data from our study showed that stents for restenosis treatment were used in 70.6% (*n* = 12) of patients, balloons in 17.6% (*n* = 3), and drugs in 11.8% (*n* = 2).

### 3.7. Relationship between Restenosis Type and Plaque Morphology

Notably, a statistically insignificant difference was obtained between the types of restenosis (χ^2^ = 7.706, df = 3, *p* = 0.052). Type of restenosis: I—11.8% (*n* = 2), II—11.8% (*n* = 2), III—52.9% (*n* = 9), IV—23.5% (*n* = 4).

Statistical analysis revealed no significant distribution between restenosis type and plaque morphology (*p* = 0.268) (Table 5). Furthermore, Kendall correlation analysis between the restenosis type and the grade of intraplaque neovascularization was performed. No significant correlation between the type of restenosis and the grade of intraplaque neovascularization was found (τ = 0.097, *p* = 0.667).

According to the angiography, the average degree of stenosis in the overall study population was 87.22% (range: 55.0% to 100.0%). In the group of stented patients without restenosis, the average degree of stenosis was 87.51%, while, in the group of stented patients with restenosis, it was 83.71%. No statistically significant difference in the degree of stenosis was found comparing both patient groups (*p* = 0.075).

## 4. Discussion

There are ideas and studies suggesting that the primary consideration of atherosclerotic plaque is an important factor in the development of a treatment strategy, although, nowadays, guidelines are based on the degree of stenosis and presence of symptoms only. Relying solely on the extent of stenosis is insufficient for accurately predicting stroke in asymptomatic patients. When evaluating patients with carotid artery atherosclerosis, ascertaining the point at which a stable atherosclerotic lesion transforms into an unstable one is a complex task, and this transition is not exclusively contingent upon plaque size. Atherosclerotic plaques have three principal components: (i) smooth muscle cells, macrophages, and T cells, (ii) an extracellular matrix including collagen elastic fibers and proteoglycans, and (iii) intracellular and extracellular lipids [23]. Plaque development is intricately associated with the process of stabilization and destabilization. Neovascularization within the atherosclerotic plaque signifies the onset of destabilization mechanisms. Angiogenesis, the formation of new blood vessels from pre-existing endothelium, is crucial in developing hemorrhage, rupture, and thrombosis within coronary atherosclerotic plaques. Consequently, this process contributes to advancing artery stenosis, ultimately leading to occlusion [24]. As a result, there has been a transition in the focus of carotid imaging toward the inclusion of plaque biology quantification in order to enhance the effectiveness of stroke risk stratification. Data and studies rely on CEUS [25] as a tool for plaque neovascularization diagnosis. Abnormal immature vessels are thought to promote plaque instability and rupture due to vascular leakage and inflammation [23]. Plaque neovascularization is not stopped after stent implantation and might have an effect on ISR. We found the association between plaque morphology and the type of restenosis.

In our study, ISR occurred in 8.1% (*n* = 18) of patients. According to other studies, ISR affects 3.5–14% of patients after CAS [26]. In our study, there were no statistically significant differences between the group of patients who smoke and have diabetes and the group comprising those who do not. Similar results were found in other studies [11,27]. In our study, we found a statistically significant difference between grades of plaque, grade 0 and 2. The study by Lingyun Jia et al. found that plaque morphology (regular or irregular), stent types (closed cell stent and open cell stent), and post-balloon dilation did not affect the occurrence of restenosis [28].

It is pertinent to note that our country has experienced the most significant increase in the age-adjusted incidence of stroke, with prevalence rates expected to rise in Lithuania [29]. Moreover, the heart score risk model indicates that Lithuania is situated within the very high-risk region for cardiovascular disease over a 5-year and 10-year period [30].

Most institutions use carotid duplex ultrasound, CTA, MRA, and digital subtraction angiography to diagnose ISR. The presence of metallic artifacts can impede the effective use of MRA for ISR diagnosis and, in turn, results in the frequent omission of this type of surveillance.

DSA is the gold standard for evaluating the carotid diseases, but, because of angiogram-related strokes and other complications, it is not routinely performed for diagnostic purposes only [31].

In conventional carotid CTA, the artifacts of the stent vary depending on the structure and characteristics of the alloy type. Also, CTA has limitations in the patients with kidney failure (which is a common condition in patients with carotid stenosis) and contrast allergies. Arteries with stents exhibit distinct biomechanical characteristics compared to native vessels, leading to heightened rigidity and stiffness, indicating reduced compliance and increased velocities [32]. The Society for Vascular Surgery has established optimal velocity threshold criteria for varying severity of ISR after CAS: PSV ≤ 104 cm/s if <30% stenosis, PSV: 105 cm/s to 174 cm/s if 30% to 50% stenosis, PSV: 175 cm/s to 299 cm/s if 50% to 70% stenosis, PSV ≥ 300 cm/s, EDV ≥ 140 cm/s, and ICA/CCA ≥ 3.8 if ≥70% stenosis [33]. However, the International Carotid Stenting Study (comparing DUS derived PSV with CTA in re-stenosis patients after CAS) found no evidence that PSV thresholds needed to be increased when diagnosing > 50% stenosis [34].

The question of the optimal timing, method, and circumstances for intervening in patients with coronary artery stenosis is a matter of ongoing debate. For asymptomatic patients with ISR less than 70%, a medical management approach involving antiplatelet agents, high-intensity statin therapy, effective blood pressure control, and tobacco cessation appear to be the most suitable course of action.

A systematic review/meta-analysis has shown a low incidence of late ipsilateral stroke, i.e., 0.8% over a 50-month period, in untreated asymptomatic patients with CAS ISR exceeding 70% [9]. For the symptomatic patients receiving maximal medical therapy, including dual antiplatelet therapy and high-intensity statin, with ISR exceeding 50% and no alternative source for ischemic stroke identified, re-intervention appears to be advisable for the majority of patients unless a palliative strategy is considered due to the severity of the stroke or presence of concurrent comorbidities. Endovascular ISR treatment can include balloon or cutting balloon angioplasty with or without stent implantation. There are no randomized trials with respect to symptomatic restenosis management: customarily, centers adopt symptomatic carotid stenosis recommendations. For patients who experiences symptoms related to the carotid territory and have a 50–99% restenosis on the same side, it is advisable to evaluate them for either a repetitive endarterectomy or endovascular treatment within 14 days of the onset of symptoms. For the individuals who have recently exhibited symptoms and have less than 50% restenosis on the same side, medical treatment is the preferred approach unless they encounter recurrent symptoms despite receiving optimal medical care (dual antiplatelet therapy and high-intensity statin therapy) [35]. Medical management is recommended for the patients who develop an asymptomatic restenosis > 70% after CAS [10]. Also, for the patients who develop focal neurological symptoms or seizures during the balloon inflation or proximal flow reversal during the primary carotid stenting, post-operative follow-ups and re-intervention for asymptomatic restenosis > 70% are recommended. Due to the symptomatic nature of the lesion, there are frequent concerns about the presence of thrombus or loose debris within the stent. Considering this, placing an additional stent in the targeted lesion might be more advantageous. Endovascular treatment strategies (restenting, balloon treatment) are usually chosen according to the type of restenosis.

Recent trials suggest that CEA and CAS have similar long-term outcomes in terms of ischemic stroke reduction for up to 10 years. The incidence of restenosis greater than 70% has been shown to be 5.8% following CEA with a median follow-up of 47 months and 10% following CAS with a median follow-up of 62 months [10]. The 10-year data from the CREST trial has shown no difference in restenosis or revascularization between CEA and CAS at 10 years [8]. Another trial has also reported restenosis and risk of stroke after stenting or endarterectomy for symptomatic carotid stenosis. Moderate (≥50%) restenosis has been found to be more common in the stenting group compared to the endarterectomy group [7].

Some limitations of this study have to be acknowledged. First, this was a retrospective study at a single medical center. Second, this study was observational. As such, the stenting methods and type of stent selected were not controlled. Undoubtedly, maximal medical therapy coupled with risk factor modification are fundamental. However, the question arises: who, if anyone, should undergo intervention? Maybe, primary diagnostic findings, such as high-grade neovascularization of the plaque, might play an important role in choosing the ISR treatment strategy. Does the nature of the CAS ISR pathology influence the decision of who to intervene upon?

## 5. Conclusions

In conclusion, there was a significant association between stent restenosis within 12–24 months after the carotid stenting procedure and an elevated grade of plaque vascularization detected by contrast-enhanced ultrasound. Moreover, one of the main factors possibly determining the grade of plaque was smoking, while diabetes did not have a significant effect. We found no association between plaque morphology and the type of restenosis.

## 6. Limitations

The data are derived from a single center, limiting generalizability.

## Figures and Tables

**Table 1:** Characteristics of the study population.

Characteristic	Value
Age, years, mean	71.7
Degree of stenosis, %, mean	83.7
Arterial hypertension, %	42.9
Dyslipidemia, %	17.2
Ischemic heart disease, %	17.4
Smoking history, %	8.1
Diabetes, %	20.8

**Table 2:** Distribution of stented patients without and with restenosis by gender.

Gender	Stented without Restenosis, % (*n*)	Stented with Restenosis, % (*n*)
Male	92.8 (141)	7.2 (11)
Female	89.9 (62)	10.1 (7)
Total	91.9 (203)	8.1 (18)

**Table 3:** Distribution of patients without and with restenosis by the grade of intraplaque neovascularization.

Grade of Intraplaque Neovascularization	Total, % (*n*)	Stented without Restenosis, % (*n*)	Stented with Restenosis, % (*n*)	*p* Value
0	51.6 (114)	55.2 (112)	11.1 (2)	<0.001
1	30.8 (68)	31.5 (64)	22.2 (4)	>0.05
2	17.6 (39)	13.3 (27)	66.7 (12)	<0.001

**Table 4:** Distribution of stented patients with restenosis grades of intraplaque neovascularization and smoking status.

Smoking Status	Grade of Intraplaque Neovascularization on CEUS, % (*n*)			Total, % (*n*)
	0	1	2	
Non-smokers	93.8 (105)	95.5 (63)	78.9 (30)	91.7 (198)
Smokers	6.3 (7)	4.5 (3)	21.1 (8)	8.3 (18)

**Table 5:** Patient distribution by restenosis type and plaque morphology.

Type of Restenosis	Grade of Intraplaque Neovascularization by CEUS, % (*n*)			Total
	0	1	2	
I	50.0 (1)	25.0 (1)	0.0 (0)	11.8 (2)
II	0.0 (0)	0.0 (0)	18.2 (2)	11.8 (2)
III	50.0 (1)	25.0 (1)	63.6 (7)	52.9 (9)
IV	0.0 (0)	50.0 (2)	18.2 (2)	23.5 (4)

## Data Availability

Data are contained within the article.
